# Supplementary material for: Crystal structure of dopamine receptor D4 bound to the subtype selective ligand, L745870
Source: eLife. 2019 Nov 21;8:e48822. doi: 10.7554/eLife.48822 (PMC6872212; doi:10.7554/eLife.48822)
Supplement: Supplementary file 1. — a. Values in parentheses present the highest resolution shell. CC1/2** (Diederichs and Karplus, 2013). b. All outliers are located in loops not involved in ligand binding. [file elife-48822-supp1.docx]

## Table 1. Data collection and refinement statistics for the mouse DRD4 and L745870 complex.

| Number of crystals | 28 |
| --- | --- |
| Resolution range (Å) | 28–3.5 (3.6–3.5)^a^ |
| Space group | P2_1_22_1_ |
| Unit cell: *a, b, c* (Å) | 46.7, 142, 146 |
| Total reflections | 73,140 |
| Unique reflections | 12,812 (1,227) |
| Multiplicity | 5.7 |
| Completeness (%) | 98.7 (95.6) |
| Mean I/sigma (I) | 3.2 (1.3) |
| Wilson B-factor (Å^2^) | 162 |
| R_merge_ | 0.230 (0.827) |
| CC_1/2_** | 0.985 (0.656) |
| No. of reflections used in refinement | 12,812 (1,210) |
| No. of reflections used for R_free_ | 636 (56) |
| R_work_/R_free_ | 0.305/0.335 |
| No. of non-hydrogen atoms:  all | 5,020 |
| proteins | 4,974 |
| ligands | 46 |
| RMS deviations |  |
| bonds (Å) | 0.004 |
| angles (°) | 1.03 |
| Ramachandran plot |  |
| favored (%) | 92.78 |
| allowed (%) | 6.47 |
| outliers (%)^b^ | 0.75 |
| Rotamer outliers (%) | 0.2 |
| Average B-factor (Å^2^)  all | 182 |
| proteins | 182 |
| ligands | 209 |

^a.^ Values in parentheses present the highest resolution shell. CC_1/2_** [66].

^b.^ All outliers are located in loops not involved in ligand binding.
